# Second to none: rationale, timing, and clinical management of clozapine use in schizophrenia

**DOI:** 10.1177/20451253231158152

**Published:** 2023-03-25

**Authors:** Mishal Qubad, Robert A. Bittner

**Affiliations:** Department of Psychiatry, Psychosomatic Medicine and Psychotherapy, University Hospital Frankfurt, Goethe University, Frankfurt am Main, Germany; Department of Psychiatry, Psychosomatic Medicine and Psychotherapy, University Hospital Frankfurt, Goethe University, Heinrich-Hoffmann-Str. 10, D-60528 Frankfurt am Main, Germany; Ernst Strüngmann Institute (ESI) for Neuroscience in Cooperation with Max Planck Society, Frankfurt am Main, Germany

**Keywords:** antipsychotics, clozapine, mortality, re-challenge, schizophrenia, treatment resistance

## Abstract

Despite its enduring relevance as the single most effective and important evidence-based treatment for schizophrenia, underutilization of clozapine remains considerable. To a substantial degree, this is attributable to a reluctance of psychiatrists to offer clozapine due to its relatively large side-effect burden and the complexity of its use. This underscores the necessity for continued education regarding both the vital nature and the intricacies of clozapine treatment. This narrative review summarizes all clinically relevant areas of evidence, which support clozapine’s wide-ranging superior efficacy – for treatment-resistant schizophrenia (TRS) and beyond – and make its safe use eminently feasible. Converging evidence indicates that TRS constitutes a distinct albeit heterogeneous subgroup of schizophrenias primarily responsive to clozapine. Most importantly, the predominantly early onset of treatment resistance and the considerable decline in response rates associated with its delayed initiation make clozapine an essential treatment option throughout the course of illness, beginning with the first psychotic episode. To maximize patients’ benefits, systematic early recognition efforts based on stringent use of TRS criteria, a timely offer of clozapine, thorough side-effect screening and management as well as consistent use of therapeutic drug monitoring and established augmentation strategies for suboptimal responders are crucial. To minimize permanent all-cause discontinuation, re-challenges after neutropenia or myocarditis should be considered. Owing to clozapine’s unique efficacy, comorbid conditions including substance use and most somatic disorders should not dissuade but rather encourage clinicians to consider clozapine. Moreover, treatment decisions need to be informed by the late onset of clozapine’s full effects, which for reduced suicidality and mortality rates may not even be readily apparent. Overall, the singular extent of its efficacy combined with the high level of patient satisfaction continues to distinguish clozapine from all other available antipsychotics.

## Introduction

With a life-time prevalence of 4.8–7.2 per 1000,^1,2^ schizophrenia is one of the most common mental disorders with a high number of disability-adjusted life years.^3^ Roughly two-thirds of all patients suffer from a recurrent or chronic course of illness,^4^ and about 30% of all patients develop resistance against standard antipsychotic treatment.^5–7^

More than 65 years after its discovery and more than 30 years after the seminal study by Kane and colleagues,^8^ clozapine remains the only effective antipsychotic drug for patients with treatment-resistant schizophrenia (TRS).^9–12^ Moreover, the superior efficacy of clozapine for crucial clinical aspects of schizophrenia beyond narrowly defined treatment resistance is very well established.^13–15^ Although these findings are reflected in all major national and international treatment guidelines,^16–20^ converging evidence from developed countries clearly indicates that clozapine remains substantially underused.^21^ It has been suggested that one major reason for this situation is a lack of sufficient training and experience regarding clozapine treatment in a considerable number of psychiatrists.^22,23^ While specific prescriber-related obstacles remain rather poorly understood,^22^ they may include a delayed detection of TRS, incomplete knowledge of clozapine’s broad beneficial effects, and an unfounded hesitance to use or maintain clozapine in accordance with guideline recommendations out of respect for its potential side-effects.^24^

Here, we review the current literature on all clinically relevant aspects of clozapine treatment with a particular emphasis on those we deem most pertinent to help rectify these issues. This includes guidance for optimal side-effect monitoring and management geared toward maximizing the number of patients, who can be treated safely with clozapine, while minimizing the overall number of treatment discontinuations. Moreover, by highlighting the current evidence for the full range of its clinical effects, we want to encourage increased use of clozapine not only in TRS but also in other patient groups, for which this unique medication can provide unmatched benefits.

## Literature selection

We based our review on a MEDLINE and Google Scholar search for all relevant topics, selecting both relevant individual clinical studies as well as meta-analyses and reviews. We included all articles that were published until November 2022. We searched for publications containing the following MeSH terms: treatment-resistant schizophrenia [AND] criteria, treatment-resistant schizophrenia [AND] treatment, treatment-resistant schizophrenia [AND] neurobiology, clozapine [AND] *xx*, with *xx* reflecting the topic we aimed to focus at that timepoint (e.g. side-effects, neutropenia, agranulocytosis, hypersalivation, pneumonia, myocarditis, re-challenge, re-challenge [AND] myocarditis, re-challenge [AND] neutropenia, withdrawal, discontinuation, pregnancy, breastfeeding elderly, metabolic syndrome, gastrointestinal side effects, sedation, mortality, pharmacokinetics, pharmacodynamics, valproate, clinical effects, clozapine-resistant schizophrenia). In addition, we manually screened reference lists of topical review articles. In cases in which we did not have any access to the full article, we contacted the study authors. Literature selection was also informed by our own clinical experience in the use of clozapine.

## Treatment-resistant schizophrenia

The international guidelines by the Treatment Response and Resistance in Psychosis (TRRIP) Working Group provide a clear consensus definition of TRS.^25^ A central criterion is the presence of persistent symptoms of at least moderate severity despite adequate standard antipsychotic treatment.^25^ Importantly, persistent symptoms do not need to cause subjective distress in patients but must have some degree of objectifiable detrimental functional impact. Moreover, pseudo-resistance due to continued use of hallucinogenic drugs or insufficient antipsychotic plasma levels needs to be excluded.^25,26^ Pseudo-resistance can also result from side-effects or comorbid medical conditions masking the clinical effects of antipsychotic treatment.^13,25^ In this context, the importance of therapeutic drug monitoring is underscored by evidence from a naturalistic clinical setting indicating that approximately 30% of patients with suspected treatment resistance should in fact be classified as ‘pseudo-resistant’ because of subtherapeutic antipsychotic plasma levels.^27–29^ Finally, a comprehensive diagnostic workup is essential to rule out other underlying disorders.

The minimal criteria for TRS encompass the following points:

1. Persistent symptoms (positive, negative, and cognitive symptoms) over at least three months of at least moderate severity causing at least moderate functional impairments. Symptoms classification and thresholds require the use of standardized, validated clinical rating scales.
2. Insufficient response to treatment with at least two different antipsychotic drugs with a minimum duration of treatment of twelve weeks (six weeks for each drug). This corresponds to a minimum chlorpromazine dose equivalent of 600 mg per day.
3. Ascertainment of sufficient treatment adherence defined as patients having taken at least 80% of the prescribed doses. To this end, at least two of the following methods need to be employed: counting pills, patient and caregiver report, and chart and record reviews. In addition, blood plasma drug levels should be monitored at least once for each antipsychotic.

**Figure 1.**
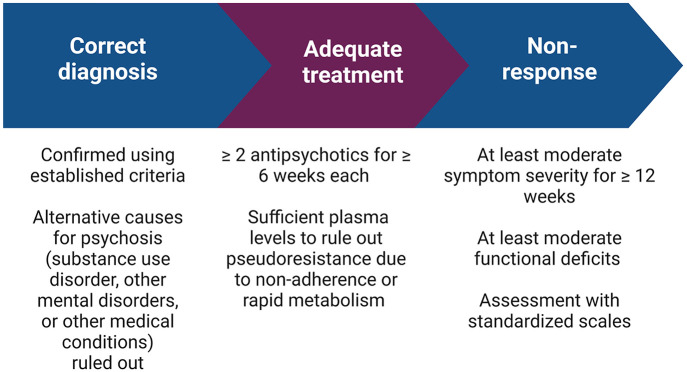
Treatment-resistant schizophrenia. Minimal criteria for the diagnosis of treatment resistance according to current TRIPP guidelines.^25^ Created with BioRender.com.

In addition to these minimal TRS criteria (Figure 1), which are most relevant for clinical practice, optimal criteria have been proposed, which are geared more toward use in clinical trials.^25^

## The neurobiology of treatment resistance

Importantly, in 70–80% of cases, treatment resistance emerges already during the first psychotic episode, highlighting the need for an early detection of this condition.^5,6,30,31^ Several risk factors for the development of TRS have been identified. These include male sex, living in a less urban area, younger age, family history of psychosis, a high load of schizophrenia risk genes, longer duration of untreated psychosis, substance abuse, and a higher number of relapses due to non-adherence.^4,32–34^ Obstetric complications, lower premorbid social adjustment, a history of suicide attempts, extended hospitalization, impaired illness insight, and comorbid personality disorders have also been associated with an increased risk for TRS.^32,34–36^

The exact neurobiological underpinnings of TRS remain elusive. Some authors distinguish between primary and secondary TRS.^7,37–40^ While primary TRS is present at illness onset, secondary TRS manifests during later stages of the disorder after an initially sufficient response to antipsychotic treatment.^7,39,40^ Dopaminergic supersensitivity has been discussed as one likely cause of secondary TRS.^7,41^ Upregulation of striatal postsynaptic dopamine D2 receptors in response to antipsychotic treatment can lead to psychotic exacerbation despite continuous treatment. Consecutive dose increases of antipsychotics can induce further receptor upregulation inducing dopaminergic supersensitivity. In general, serotonin dysregulation and inflammation as well oxidative stress have been proposed to be involved in the pathophysiology of TRS.^7^ There is also converging evidence that abnormalities in glutamatergic neurotransmission might contribute to the emergence of treatment resistance,^41^ which would also be compatible with the existence of a normodopaminergic subtype of schizophrenia.^7,37,38^ A higher genetic load for schizophrenia also appears to increase the risk for TRS.^7,33,42,43^

## Clinical effects of clozapine

Unequivocal evidence supports the superior efficacy of clozapine for the reduction of positive symptoms and global psychopathology in TRS compared with other antipsychotics.^8,12,44–46^ Patients treated with clozapine also show improved treatment adherence^11^ and reduced rehospitalization rates.^47–52^ It is crucial to emphasize that there is no evidence for a comparable efficacy of antipsychotic polypharmacy, that is, the combination of two non-clozapine antipsychotics.^16^ Therefore, offering clozapine should always take precedence when treating patients with TRS.

Importantly, the beneficial effects of clozapine go far beyond positive symptoms. Clozapine is among the most effective antipsychotics for improving negative symptoms.^14,46,53^ It also shows a similar level of efficacy against depressive symptoms,^14^ which constitute a common, independent risk factor for suicidality in schizophrenia.^54^ Accordingly, clozapine leads to a stronger reduction of both suicidal behavior^55–57^ and suicide mortality compared with other antipsychotics.^15^ Some guidelines therefore explicitly recommend clozapine for persistent suicidality independent of treatment resistance.^16–20^ Compared with other antipsychotics, clozapine also shows superior efficacy in reducing aggressive and violent behavior.^58–62^ Furthermore, clozapine lowers the risk of developing a substance use disorder (SUD),^52^ and also reduces relapse rates in patients with a comorbid SUD.^52,63^

Response rates to antipsychotic treatment in drug-naïve patients are estimated at 75%.^64,65^ Conversely, response rates to a second trial with a standard antipsychotics are considerably lower, ranging between 20% and 45%.^64,65^ Estimates for overall response rates range between 40% and 60%.^66,67^ Clinical efficacy of clozapine depends crucially on early treatment initiation.^66,68–70^ Response rates for treatment initiation within the first 2–3 years after establishing treatment resistance can reach up to 80%.^64,66,71^ For later treatment initiation, response rates can be as low as 30%.^66^ Combined with the clear evidence for a predominantly early onset of treatment resistance, these findings underscore the vital importance of offering and starting clozapine early.

## Pharmacodynamics

Clozapine is an antagonist at all dopamine-receptor subtypes (D1–D5).^72^ Among them, the antipsychotic effects of clozapine appear to be primarily mediated *via* D2 receptor antagonism.^73^ In this regard, clozapine mirrors other antipsychotics, but its superior efficacy for positive symptoms appears to be the result of additional pharmacological properties. Even after decades of clinical use, the neurobiological mechanisms underlying the broad superior clinical efficacy of clozapine remain elusive. Currently, effects in the glutamatergic^74–77^ as well as the GABAergic system are discussed as likely explanations.^78–80^ The pleiotropic effects of clozapine, however, also encompass neurobiological systems not directly related to neurotransmission,^37,75,81–83^ but their relevance remains unclear.

By comparison, the properties underlying clozapine’s side-effect profile are relatively well established. The nearly complete absence of extrapyramidal symptoms is most likely attributable to rapid dissociation of clozapine from striatal D2-receptors.^84^ Antagonism at serotonergic 5HT2C- and 5HT2A-receptors^85,86^ and at histaminergic H1-receptors is implicated in clozapine-associated weight gain.^85^ Antagonism at H1-histaminergic receptors^87,88^ as well as agonism at gamma-aminobutyric acid (GABA) B receptors have been implicated in sedation.^80^ Serotonergic antagonism appears to be involved in clozapine-associated obsessive compulsive symptoms.^89,90^ Clozapine’s unique muscarinic profile is responsible for several highly relevant side effects. Agonistic effects at M4-receptors are the primary cause of hypersalivation. Conversely, antagonism at M2-receptors is implicated in clozapine-induced gastrointestinal hypomotility (CIGH). Anticholinergic mechanisms might also worsen symptoms associated with cognitive decline, cause delirium, and urinary retention.^91^ In addition, clozapine’s antagonistic properties at adrenergic receptors have been linked to nocturnal enuresis, hypotension, and hypersalivation.^92,93^

## Pharmacokinetics and interactions

Clozapine’s half-life is approximately 14 h.^94,95^ Its metabolism is influenced by several factors including hormones like estrogens, concurrent medication, smoking,^96^ sex, with higher blood plasma levels in females,^97,98^ and age.^96,99–102^ Ethnicity can also have an effect, with people of Asian descent generally reaching sufficient clozapine plasma levels at lower doses than Caucasians.^100^

The following cytochrome P450 (CYP)-enzymes are mainly involved in clozapine metabolism: CYP1A2 (30%), CYP2C19 (24%), CYP3A4 (22%), CYP2C9 (12%), and CYP2D6 (6%).^103^ Among them, CYP1A2 induction or inhibition can lead to clinically relevant changes in plasma clozapine levels.^94,95^ Inhibitors include caffeine,^104^ and C-reactive protein (CRP),^105–107^ which can be triggered by infections. This is underscored by recent reports of toxic plasma clozapine levels during SARS-CoV-2 infections.^108,109^ Oral contraceptives containing estrogens also inhibit CYP1A2 and CYP2C19 enzyme activity leading to clinically relevant plasma level increases.^110,111^ Polycyclic aromatic hydrocarbons (PAHs) contained in cigarette smoke are the most relevant CYP1A2 inducers.^112,113^ Importantly, after abrupt smoking cessation, enzyme activity typically normalizes within three days,^112^ which can lead to toxic plasma clozapine levels.^113–121^ Notably, nicotine patches and e-cigarettes are not associated with a comparable interaction risk.^28,122–125^

Optimal plasma clozapine levels for the treatment of TRS fall in the range of 350–600 µg/L.^126,127^ Plasma clozapine levels above 600 µg/L increase the risk for side-effects considerably.^128^ Plasma clozapine levels above 1000 µg/L are considered toxic, are associated with an at least two-fold increase in mortality risk,^129^ and require immediate dose reduction and intensified pharmacovigilance. Plasma clozapine levels above 2000 µg/L are acutely life-threatening.^130,131^ Notably, clozapine intoxication is associated with a delayed plasma peak due to clozapine’s extensive enterohepatic circulation and its induction of gastrointestinal hypomotility.^132^ In general, international guidelines strongly recommend regular monitoring of plasma clozapine levels to increase both patient safety and response rates.^28,29^

In rare cases, rapid metabolism of clozapine due to yet unknown causes may preclude reaching sufficient plasma clozapine levels.^133–135^ Here, augmentation with low doses of fluvoxamine, a strong CYP1A2 inhibitor, should be considered.^136–138^ A total of 25–50 mg of fluvoxamine can raise plasma clozapine levels five- to ten-fold within 2–4 weeks^139–141^ and also triple clozapine’s half-life.^142^ Consequently, frequent screening for side effects and therapeutic drug monitoring are crucial during augmentation with fluvoxamine.^28,29^

In summary, there are several relevant pharmacokinetic and pharmacodynamic interactions clinicians need to be aware of (Tables 1 and 2). When addressing such interactions, switching to safer alternatives for interacting drugs wherever possible should be the primary strategy. Discontinuation of clozapine should only be considered as a last resort.

**Table 1.** Pharmacokinetic interactions.

CYP inducers	CYP inhibitors
Omeprazole^143^	Selective serotonin re-uptake inhibitors (SSRIs; e.g. fluvoxamine und fluoxetine; sertraline in high doses)^104,143^
Carbamazepine^128,144^	Quinolone antibiotics (e.g. ciprofloxacin)^104,143^
St. John’s wort^128^	Macrolide antibiotics (e.g. erythromycin)^143^
PAH^143^	Caffeine^104^
	Ethinyl estradiol^104,143^
	Propranolol^145^

CYP, cytochrome P450; PAH, polycyclic aromatic hydrocarbon.

**Table 2.** Pharmacodynamic interactions.

Side-effects	Most relevant medications
Hypotension	Tricyclic antidepressants (TCA), antihypertensive medication (e.g. propranolol, ACE inhibitor)^73,146^
Sedation	Benzodiazepines^73^
Anticholinergic gastrointestinal side-effects	TCA, anticholinergic drugs^73,92,147^
Other anticholinergic side-effects (e.g. delirium)	TCA, anticholinergic drugs, opioids, antihistaminergic drugs^73,92,94,147^
Hematological side-effects	Carbamazepine, metamizole, TCA, mirtazapine, bupropion, valproate, carbimazole, cytostatic drugs, chloramphenicol, sulfonamide, co-trimoxazole^73,94,148–150^
Reduction of seizure threshold	Bupropion, lithium, TCA^73,94,151–153^
QT prolongation	Macrolide antibiotics (e.g. erythromycine), quinolone antibiotics (e.g. moxifloxacin), TCA^73,154–156^
Myocarditis	Valproate^94,157–159^

ACE, angiotensin-converting enzyme.

### Clozapine metabolites

Clozapine is mainly demethylated to *n*-desmethylclozapine (norclozapine) and oxidized to clozapine-*n*-oxide.^95^ In contrast to clozapine-*n*-oxide, norclozapine is pharmacologically active. Compared with clozapine, norclozapine shows diverging effects on dopaminergic and muscarinic receptors^98,160^ and also affects serotonergic receptors among others.^161–164^ While it has no antipsychotic properties, norclozapine appears to contribute to the overall side-effect burden including sedation, hypersalivation, constipation, metabolic complications, and seizures.^163^

Recently, the clozapine:norclozapine ratio has received growing attention.^98,136,165^ Based on clinical observations, the optimal clozapine: norclozapine ratio is deemed to be around two. Higher values are indicative of a non-trough blood sample, a recently missed dose, or decreased CYP1A2 enzyme activity. Lower values appear to be indicative of increased CYP1A2 enzyme activity.^136,165,166^ Other authors, however, recommend using the total clozapine C/D ratio, with C representing the sum of the clozapine and norclozapine trough steady-state plasma concentration and D representing the daily dose of clozapine.^98^

## Side effects of clozapine

**Figure 2.**
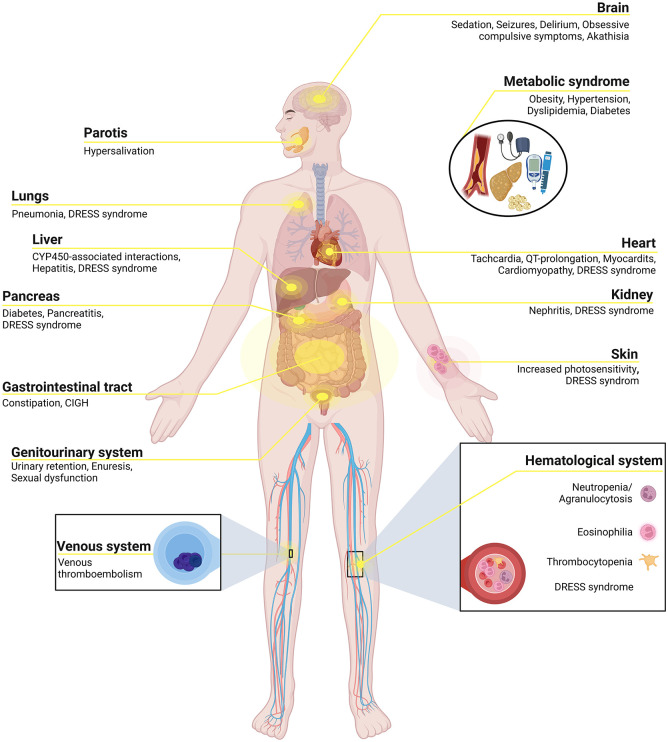
Clozapine-associated side effects. Overview of the clinically most relevant side effects encountered in patients receiving clozapine. Adapted from “Human Internal Organs”, by BioRender.com (2022). Retrieved from https://app.biorender.com/biorender-templates (accessed on 16th November 2022).

Owing to the relatively high side-effect burden associated with clozapine (Figure 2 and Table 3), extensive pharmacovigilance as well as early and consistent management of side-effects are of particular importance.^167^ Frequent consultation of both patients and their families is an important element of this strategy.^101^ In addition to clozapine’s broad antagonistic and agonistic effects on key neurotransmitter systems outlined above, immunomodulatory effects, which might partly explain clozapine’s unique efficacy, have also been implicated in adverse drug reactions (ADRs) including eosinophilia, myocarditis, pancreatitis, and nephritis.^167^ Side-effect risk decreases with slower initial dose titration regimes.^101^ This is particularly important in light of evidence for an association between rapid initial dose escalation and risk for both myocarditis and neutropenia.^92^ Owing to the clear dose dependency of some side effects (Table 3), dose reductions should be attempted first whenever feasible before considering other options.^101^

**Table 3.** Management of clozapine-associated side effects.

Side-effect	Monitoring and diagnostics	Prevention and management
Metabolic syndrome* (obesity, hypertension, dyslipidemia, diabetes)^168–170^	• Baseline-screening: weight/BMI, lipid profile, HbA1c ○ BMI/weight: monthly 1st/2nd/3rd/6th month, then yearly^171–173^ ○ Lipid profile: quarterly^174,175^ ○ Diabetes screening: quarterly^94^	Non-pharmacological treatment: • Lifestyle changes, psychotherapy^176,177^ Pharmacological treatment: • Obesity/weight gain: as early as possible; mandatory in cases of weight gain of ⩾7% and in cases with BMI ⩾25: ○ Metformin^13,16,178,179^ - contraindication for metformin: eGFR of <30 ml/min - frequent monitoring of vitamin B_12_ level^177^ ○ Alternative options: GLP1-receptor agonists,^180^ topiramate^181–184^ • Hypertension: antihypertensive drugs^176,185,186^ • Dyslipidemia: statins^176^
Hypersalivation (increases risk for pneumonia)^187–191^	• Regular clinical assessment using standardized rating scales, for example, the Drooling severity scale or Nocturnal Hypersalivation Rating Scale^192,193^	• Sugar free chewing gum during the day to promote saliva swallowing; elevation of upper body during the night^193^ • Pirenzepine (25–100 mg/d); Cave: anticholinergic load^16,94,191^ • Alternative options: atropine drops s.l., ipratropium bromide s.l.^191^ • Botulinumtoxin injection (incobotulinum toxin A) into the parotid and submandibular glands^16,189,194–198^
Sinus tachycardia^199,200^	• Regular clinical assessment including ECG^126^ • Diagnostics: 12 channel ECG, 24-h Holter monitoring; consider stress ECG, TTE, laboratory tests according to established guidelines^201,202^	• Treatment with ivabradine (no depressogenic effects reported) or cardio-selective beta blockers (Cave: hypotension, bronchospasm, depressogenic)^16,94,199,203,204^
Constipation, CIGH*^205,206^	• Regular clinical assessment^94,101,167^	• Physical activity, sufficient fluid intake, fiber-rich diet^16,94,207,208^ • Discontinue non-essential drugs that increase anticholinergic load^209,210^ • Treatment with laxatives^207,208,211^
Hematological side-effects^212,213^	• Complete differential blood count: at baseline, once weekly (1st–18th week), then monthly; in case of discontinuation continue monitoring for 4 weeks^73,94,214^ • Leukopenia (white blood cells [WBCs] <4/nl) *versus* CIN (ANC <1.5/nl) *versus* CIA (ANC <0.5/nl)^73,214–216^ ○ In cases of WBC 3–3.5/nl and ANC 1.5–2/nl: Monitoring twice per week^94,217–221^ • Eosinophilia >3/nl: search for CIM, pancreatitis, DRESS syndrome, CIA • BEN: does not constitute a contraindication; consider expanding monitoring^221–225^ • Most hematological changes are only transient^226^	• Confirmed CIA (ANC <0.5/nl): discontinue clozapine, infection prevention, consider administration of G-CSF/GM-CSF^16,217–221^• Thrombocytopenia <50/nl: discontinue clozapine temporarily^227,228^ • Eosinophilia >3/nl: discontinue clozapine temporarily; do not restart clozapine unless eosinophil count <1/nl^73,94,217,229–233^
Sedation*^88,190,234^	• Regular clinical assessment • Most commonly transient in nature^88,190,234^	• Titration to lowest effective dose^16,190,234^ • Minimize daytime doses^190,234^ • Avoid concomitant sedating drugs^94,190,234^
Myocarditis, cardiomyopathy^235,236^	• Highest risk during the first 4 weeks of treatment • Risk factors: rapid dose titration (>25 mg/d), higher age, concomitant valproate^237^ • Mandatory clinical, electrophysiological, and laboratory monitoring at baseline and during the first 8 weeks of treatment: heart rate; ECG; TTE (if available); CRP, troponin, CK, BNP, full blood count^94,238,239^ • Clinical signs: cardiac symptoms/gastrointestinal and urogenital disturbances including non-specific flu-like symptoms, dyspnea, diarrhea, fever; increased heart rate by ⩾20–30/min, signs of reduced left ventricular function^94,239^ • Cardiac monitoring signs: unspecific changes in ECG; TTE: changes in pericardial effusion, cardiac wall motion abnormalities^240^ • Laboratory signs: CRP, troponin, CK, BNP: ↑, eosinophilia (delayed)^94,239,241^	• Mandatory discontinuation of clozapine in case of CRP level increases of more than 10× upper limit normal (ULN) or troponin level increases of more than 2× ULN^212,235,236,242^ • Cardioprotective treatment: ACE-inhibitors and beta blockers^243^ • In severe cases, transfer to intensive care unit^94,244^ • Symptoms improve within 5 days of treatment termination; most common course: *restitutio ad integrum*,^94,242^ cardiomyopathy is a potential complication
QT prolongation*^141,154–156^	• Frequent ECGs: weekly for the 1st month, afterwards at least quarterly^94,141,154–156,245^ • Use corrected QT-time with appropriate formula (e.g. *Fridericia*)^141^	• Avoid concomitant drugs causing QT prolongation whenever possible^141,246^ • Slow dose titration^141,246^ • Avoid hypokalemia and hypomagnesemia^245,247^ • Consider oral supplementation of magnesium^141^ • Search for signs of CIM^141^
Seizures*^163,248–250^	• EEG: at baseline, after 3 months, afterwards every 6 months^94^ • Risk factors: higher doses, rapid dose titration, history of seizures or head trauma, concomitant medication or compounds resulting in pharmacodynamic or pharmacokinetic changes (e.g. lithium, smoking cessation), physical illness (e.g. hyponatremia), substance abuse^73,94,104,162,249^ • Pre-existing and sufficiently medically controlled epilepsy does not constitute a contraindication^94,250^	• Consider clozapine dose reduction by about 50% • Combine with anticonvulsant medication: lacosamide (Cave: neutropenia), gabapentine, lamotrigine;^94,151,249–251^ avoid valproate (risk factor for CIM)
Obsessive compulsive symptoms^252,253^	• Regular clinical assessment • Largely independent of dose and treatment duration^89^	• Clozapine dose reduction • CBT, SSRI, consider combination with aripiprazole^89,94^
Akathisia*^94,254–256^	• Regular clinical assessment • Cave: can be present without overt motor signs^257^	• Dose reduction^92,258,259^ • Propranolol (30–120 mg) – Cave: drug–drug interaction *via* CYP450-enzymes^145,255,260^ • Mirtazapine (7.5–15 mg)^255,260^
Pneumonia^261^	• Risk factor: hypersalivation, sedation, older age, male sex, concomitant medication (e.g. *via* promoting sedation)^261–263^	• Prevention: early treatment of hypersalivation^153,264,265^ • In case of pneumonia: adjust clozapine dose, increase frequency of monitoring and consider increased risk for interactions associated with CYP450 enzymes (e.g. CRP, antibiotics)^94,266^ • Ensure sufficient respiratory disease vaccination status (influenza and SARS-CoV2)
Other clinically relevant side effects: hepatitis, nephritis, pancreatitis,^267,268^ delirium*, enuresis, NMS,^93,94,167,269,270,271^ DRESS syndrome,^230,272^ venous thromboembolism,^212,273^ diabetic ketoacidosis, hyperosmolar coma^212,274^	• Clinical assessment, laboratory parameter^275^ • Cave: delirium associated with high clozapine doses and with abrupt clozapine discontinuation^275,276^ • NMS: rare (clozapine is the drug of first choice after NMS)^269,271,277^ • Pancreatitis: screen for signs of exocrine and endocrine pancreas insufficiency • DRESS syndrome: assess laboratory parameters frequently (especially eosinophils, lymphocytes);^230,272^ risk factor: combination with lithium, anticonvulsants (including valproate)	• Enuresis: avoid late fluid intake, continence training, desmopressin^94,270,278^ • Hepatitis, pancreatitis, nephritis: rare; discontinue clozapine, initiate specific treatment^94,268,279^ • Delirium: pause clozapine,^73,275,280^ treat delirium^94^ • DRESS syndrome: discontinue clozapine; initiate symptomatic treatment (e.g. antipyretic, antihistaminergic, immunosuppression with steroid/intravenous immunoglobulins)^230,272^

ACE, angiotensin-converting enzyme; ANC, absolute neutrophil count; BEN, benign ethnic neutropenia; BMI, body mass index; BNP, brain natriuretic peptide; CBT, cognitive behavioral therapy; CIA, clozapine-induced agranulocytosis; CIGH, clozapine-induced gastrointestinal hypomotility; CIM, clozapine-induced myocarditis; CIN, clozapine-induced neutropenia; CK, creatine kinase; CRP, C-reactive protein; CYP, cytochrome P450; DRESS, drug reaction with eosinophilia and systemic symptom; ECG, electrocardiography; EEG, electroencephalography; G-CSF, granulocyte colony-stimulating factor; GLP1, glucagon-like peptide-1; GM-CSF, granulocyte-macrophage colony-stimulating factor; NMS, neuroleptic malignant syndrome; SSRI, selective serotonin re-uptake inhibitor; TTE, transthoracic echocardiogram.

* Dose-dependent side-effect.

### Hematological side effects

Not least for historic reasons,^281^ clozapine is closely associated with agranulocytosis and other, less serious forms of neutropenia. An absolute neutrophil count (ANC) of 1–1.5/nl is referred to as mild neutropenia while ANCs of 0.5–1.0/nl are referred to as moderate neutropenia. ANCs of <0.5/nl constitute severe neutropenia.^282^ ANCs below 0.5/nl are also typically referred to as agranulocytosis.^215,283,284^ However, strictly speaking agranulocytosis requires near absence of neutrophils, that is, ANCs below 0.1/nl.^283,285^ The clinical syndrome of agranulocytosis is commonly associated with a triad of symptoms encompassing fever, mouth ulcers, and sore throat.^215^ Pragmatically equivalating severe neutropenia and agranulocytosis is motivated by the substantial risk for opportunistic infections associated with ANCs below 0.5/nl,^215^ which triggers several clinical actions discussed in detail below. For pragmatic purposes, we will therefore likewise refer to ANCs of 1.5–0.5/nl as clozapine-induced neutropenia (CIN) and to ANCs of <0.5/nl as clozapine-induced agranulocytosis (CIA).

Owing to an increased risk for CIN and CIA, frequent blood cell counts are mandatory throughout treatment.^212,281,286^ This procedure has reduced risk of death from CIA to less than 1 in 10,000 patients.^238^ Risk for CIN and CIA is estimated to be 3% and 0.4–0.7%, respectively.^217,287^ Although CIN or CIA can occur at any time during treatment,^288,289^ the highest incidence rates have been observed during the first 6–18 weeks of treatment (49% cases of neutropenia, 82% cases of agranulocytosis) with a clear subsequent risk decrease after six months.^73,213,217,287,290^ This should be considered when a comorbid somatic disorder necessitates treatment with a drug also linked to blood dyscrasia. Whenever possible, treatment with such drugs should be initiated after the critical first six months.^213^

Currently, safety thresholds for neutrophil counts during clozapine treatment vary slightly across health systems. Importantly, discontinuation should require clear evidence for a downward dynamic of the neutrophil count,^219,220,291^ as cases of transient neutropenia under clozapine treatment have also been observed.^292^ Moreover, an immediate thorough search for other causes of blood dyscrasia is essential, as this would have important implications for a potential re-challenge of clozapine.^217,218,287^ Important causes include concomitant medication – including antibiotics^273,293^ and psychotropic compounds like carbamazepine and valproate^294,295^ – or viral infections.^296^

Confirmed CIN and CIA stipulate immediate discontinuation of clozapine. In addition, CIA requires the administration of granulocyte colony-stimulating factor (G-CSF). Moreover, further actions to prevent and treat infections, for example, administration of antibiotics and protective isolation, might be necessary.^297–299^

**Figure 3.**
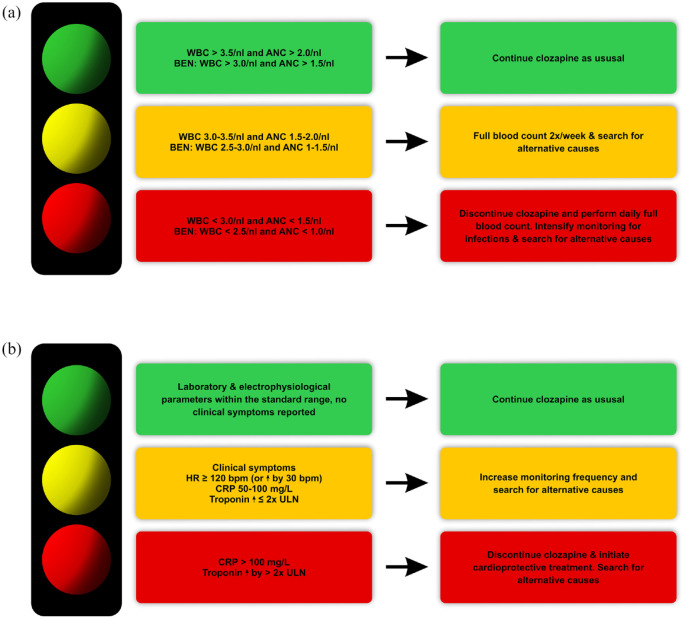
Screening and management of clozapine-induced neutropenia (CIN) and clozapine-induced myocarditis (CIM). (a) Color code categorization of CIN screening parameters and required action. Absolute neutrophil counts within the green range do not require any action except regular monitoring. ANCs within the yellow range require intensified full blood monitoring and searching for an alternative cause. ANCs within the red range necessitate immediate discontinuation of clozapine, daily monitoring of full blood count, and searching for an alternative cause. (b) Color code to categorization of CIM screening parameters and required action. Results within the green range do not require any action except regular monitoring. Results within the yellow range require intensified monitoring and searching for an alternative cause. Results within the red range necessitate immediate discontinuation of clozapine, daily monitoring, initiation of cardioprotective treatment, and searching for an alternative cause.

In cases of mild neutropenia even before treatment onset, benign ethnic neutropenia (BEN) is an important differential diagnosis, which does not constitute a contraindication.^221–225^ Safety thresholds for patients with a confirmed diagnosis of BEN are lower (Figure 3(a) and Table 3).^300^ Pseudo-neutropenia resulting from physiologically reduced neutrophil counts due to higher cortisol levels in the morning should also be considered.^217^

The pathophysiology of CIN and CIA remains poorly understood, but an autoimmune mechanism appears most likely. Eosinophilia is another important but rare hematological side effect. Importantly, eosinophilia does not warrant permanent discontinuation of clozapine.^212,301^ Treatment can be restarted at eosinophil counts below 1/nl. Eosinophilia, however, should prompt a search for other clozapine-induced ADRs including CIA, clozapine-induced myocarditis (CIM), pancreatitis, and drug reaction with eosinophilia and systemic symptom (DRESS) syndrome.^227,302^

Notably, a recent longitudinal study revealed an increased risk for hematological malignancies in patients receiving clozapine.^303^ This risk, however, is smaller than the reduction of all-cause mortality associated with clozapine. Moreover, mortality rates in clozapine users diagnosed with a hematological malignancy were lower compared with patients treated with nonclozapine antipsychotics.^303–305^ Therefore, while these findings necessitate increased vigilance regarding signs of hematological malignancy in clozapine users, they do not undermine the general case for clozapine.^304,305^

### Cardiac side effects

CIM is among the most important and yet underdiagnosed side effects. CIM risk is the highest during the first 4 weeks after treatment initiation,^306,157^ but also during re-exposure following a first successful trial.^94^ Moreover, rare cases of CIM after long-term treatment of more than a decade have also been observed.^73,307,308^ Compared with CIN and CIA, the incidence of CIM is noticeably higher.^157,309^ The timely detection of CIM might be impeded by its often unspecific clinical presentation, which can include flu-like symptoms like fever, dyspnea, myalgia, and vague complaints of fatigue and malaise.^242,309,310^ Patients might also experience symptoms reflecting cardiac involvement such as chest pain, hypotension, palpitation, tachycardia, and peripheral edema.^242,309,310^ These more specific symptoms, however, are by no means mandatory. By contrast, there have been reports of cases solely presenting with gastrointestinal and urogenital disturbances like diarrhea, dysuria, and vomiting.^241,242,310,311^ Hence, frequent screening for clinical, electrophysiological, and laboratory signs of CIM is mandatory.^94,147,167,241^ Electrocardiographic (ECG) findings in CIM are characterized by non-specific alterations such as T-wave inversion and ST elevation or depression.^242,312^ Transthoracic echocardiogram (TTE) might reveal left ventricular impairments and pericardial effusion, while cardiac magnetic resonance imaging (MRI) can provide more direct evidence for myocardial inflammation.^240,313^ In addition, endomyocardial biopsy can be performed to rule out viral myocarditis.^314^ Mandatory laboratory screening at baseline and during the first 8 weeks of treatment encompasses troponin, CRP, creatine kinase (CK), and brain natriuretic peptide (BNP)^13,242,310,312,315^ are highly sensitive markers for CIM.

Importantly, while monitoring of clinical symptoms is important to inform CIM diagnosis, confirmation of a suspected CIM should rely primarily on objective parameters, that is, laboratory parameters exceeding pre-specified thresholds (Figure 3(b) and Table 3), to prevent premature and unnecessary discontinuation but also to ensure patient safety in cases of unspecific clinical symptoms.^310^ Clearly established CIM necessitates immediate termination of clozapine,^246,310^ strict avoidance of major physical activity,^244^ and initiation of a cardioprotective pharmacotherapy with a beta-blocker and an angiotensin-converting enzyme (ACE) inhibitor.^73,241,242,246,316^ Serious cases of CIM might require treatment in an intensive care unit.^4,244,317^ Severe outcomes of CIM – typically as a consequence of delayed or missed diagnosis and treatment^318^ – include ventricular arrhythmia, persistent heart failure, and sudden death.^158,244,246,312^ Importantly, early intervention increases the chance for a *restitutio ad integrum*,^242^ underscoring the relevance of CIM screening during the initial titration of clozapine.^318^

Based on its early onset and titration speed dependency, a hypersensitivity reaction is discussed as a likely pathophysiological mechanism of CIM,^235,244,246,319–323^ but current evidence remains inconclusive.^157^ Thus far, no reliable predictors for an individual’s CIM risk have been established. Several risk factors for CIM, however, have been identified,^157^ chiefly among them concurrent treatment with valproate.^157,237,324^ Consequently, valproate should not be prescribed concurrently with clozapine, neither for treatment of any residual psychopathology nor for seizures. There is also preclinical and clinical evidence that rapid titration increases the risk of CIM.^237,319,325^ This suggests that people with slow clozapine metabolism might be at a higher risk for CIM. Higher age and higher clozapine doses also appear to constitute risk factors.^157,326^

Cardiomyopathy constitutes another potential cardiac side effect with reported incidence rates ranging between 0.02% and 1%.^215,222^ Importantly, undetected and self-limiting myocarditis has been implicated as a likely cause.^198,215,222^ Hence, prevention and adequate management of CIM might also reduce the risk of clozapine-associated cardiomyopathy.

### Gastrointestinal side effects

The most common gastrointestinal side-effect is constipation, which occurs in up to 60% of patients.^327–329^ Furthermore, at least 50% of patients show unambiguous evidence of CIGH in colonic transit studies.^327^ Potentially severe consequences of CIGH include dysphagia, ileus, intestinal obstruction, bowel ischemia, and megacolon,^328,329^ which are associated with a considerable mortality rate.^330^ Importantly, the prevalence of CIGH is markedly higher than the prevalence of CIM and CIA.^329,331^ CIGH arises primarily due to the anticholinergic and antiserotonergic effects of clozapine^176^ and shows a clear dose dependence.^167,327^ Polypharmacy, which adds to clozapine’s inherent anticholinergic load or otherwise affects gastrointestinal motility, constitutes a risk factor for CIGH.^209,210^ As a first step, prevention and management of CIGH requires lifestyle and dietary modifications, that is, physical exercise, sufficient fluid intake, and fiber supplementation. As a second-line approach, current guidelines recommend specific pharmacological treatment including laxatives (first line: osmotic laxatives, second line: stimulant laxatives), prosecretory agents, and serotonergic agonists.^207,208,211^

### Metabolic side effects

Irrespective of medication, patients with schizophrenia have an increased risk for developing a metabolic syndrome, encompassing hypertension, dyslipidemia, obesity, and diabetes.^168–170^ For clozapine, the prevalence of metabolic syndrome is estimated to be approximately 50%.^332,333^ Weight gain represents a crucial risk factor for developing metabolic syndrome^334^ and an important psychological stressor.^177,335–337^ Moreover, it reduces quality of life,^335^ treatment adherence,^337,338^ and contributes to cognitive dysfunction.^339–341^ Importantly, weight gain typically occurs during the early stages of antipsychotic treatment.^342,343^ More than two-thirds of patients show a gain of more than 7% of their baseline weight during the first year of treatment.^177,344,345^ This underscores the necessity of frequent monitoring of body weight and metabolic parameters and of early weight-stabilizing interventions (Table 4).^333,346^ Regular physical exercise, a Mediterranean diet, and smoking cessation to decrease cardiovascular risk are the primary recommendations to counteract weight gain.^226,347^ Among pharmacological interventions, adjunctive metformin shows a good safety profile and the best efficacy for stabilizing weight gain.^177,178,348,349^ Consequently, the first guideline for metformin use during antipsychotic treatment advocates a routine early initiation in addition to behavioral interventions to most effectively minimize weight gain and cardiometabolic risk.^177^ On average, treatment with metformin leads to a weight loss of about 3.2 kg. As the feasibility of reversing weight gain remains limited, weight stabilization should be the primary goal. The recommended daily dose of metformin is 2000 mg when tolerable. Gastrointestinal issues such as nausea, diarrhea and vomiting as well as lactic acidosis are the most relevant side effects. Before and during treatment with metformin, renal function and vitamin B_12_ level should be monitored routinely.

**Table 4.** Management of clozapine-resistant schizophrenia.

Predominant persistent symptoms	Recommended augmentation strategies^350,351^
Positive symptoms and combined positive and negative symptoms	• Intensify CBT and psychosocial interventions • ECT • Add-on treatment with amisulpride or aripiprazole
Negative symptoms	• Intensify CBT and psychosocial interventions • Add-on treatment with antidepressants (i.e. SSRI) • Add-on treatment with mood stabilizer (avoid valproate) • ECT
Suicidality	• Mood stabilizers (lithium or lamotrigine, avoid valproate) • Antidepressant • ECT
Aggressive behavior	• Add-on treatment with mood stabilizer or another antipsychotic drug

CBT, cognitive behavioral therapy; ECT, electroconvulsive therapy; SSRI, selective serotonin re-uptake inhibitor.

More recent findings indicate that glucagon-like peptide-1 (GLP1) receptor agonists could also be effective for mitigating clozapine-associated metabolic comorbidity.^180^ Topiramate can also be considered as its efficacy appears to be comparable with metformin.^181–184,352^ Recently, samidorphan – an opioid receptor antagonist – was introduced as an adjunctive treatment for olanzapine-associated weight gain.^353,354^ This might be also a promising approach for clozapine.

Treatment of manifest diabetes in patients treated with clozapine should closely follow current guidelines including the use of insulin when indicated.^355,356^ Adequate antidiabetic treatment substantially reduces cardiovascular risk and the risk for ketoacidosis or hyperosmolar coma.^274^ Importantly, pre-existing diabetes mellitus in patients with TRS does not constitute a contraindication for clozapine treatment,^357^ but rather requires intensified antidiabetic treatment. This is underscored by converging findings indicating that, for clozapine, the risk for diabetes is not excessive compared with second-generation antipsychotics as a whole, but rather falls in the higher range within this class of drugs.^343,358^ In conclusion, it needs to be emphasized that strict management of metabolic side effects can notably improve the associated cardiovascular risk factors^359^ and that overall clozapine treatment is actually associated with a relevant reduction of cardiovascular mortality.^15,46^

### Hypersalivation and pneumonia

Clozapine-induced hypersalivation (CIH) constitutes a frequent side-effect with incidence rates ranging between 30% and 92%, which typically occurs early during treatment and shows no clear dose dependence.^187,360^ Agonism at muscarinic M4 receptors and antagonism at α2-adrenergic receptors have primarily been implicated in its etiology.^188,361,362^ Predominantly, patients report nightly hypersalivation.^187^ CIH can cause psychological distress due to embarrassment and social stigma, as well as lead to somatic adverse events like skin irritation, parotitis, and aspiration pneumonia.^73,194^ Management of CIH commonly requires early pharmacological intervention, usually with anticholinergic, that is, antimuscarinic, drugs. Evidence for their efficacy remains limited, however. Moreover, systemically acting drugs such as pirenzepine increase the overall anticholinergic load, raising the risk for CIGH.^209,210,363^ Sublingual administration of atropine eye drops^73,364,365^ and regular botulinum toxin injections into the parotid and submandibular glands can avoid this problem.^195,366^ Before recommending treatment with sublingual atropine eye drops, prescribers need to ensure that patients can adequately follow instructions regarding the finely dosed topical application to prevent accidental ingestion of relevant amounts of fluid. Importantly, CIH increases the risk of clozapine-associated pneumonia *via* salivary aspiration.^367^ In this context, it is crucial to acknowledge the higher mortality rates of patients with schizophrenia due to pneumonia and increased rates for additional pulmonary diseases including chronic obstructive pulmonary disease.^368^ Importantly, pneumonia elevates CRP levels, thus decreasing CYP1A2 enzyme activity and increasing plasma clozapine levels.^105,369–372^ This underscores the necessity for preventive measures for clozapine-associated pneumonia. Furthermore, health care professionals should ensure a sufficient vaccination status of their patients for respiratory diseases including COVID-19 and influenza in order to reduce mortality rates.^368,373^

### Sedation

Clozapine-induced sedation (CIS) ranks among the most commonly reported side-effects.^189,374–376^ It shows a clear dose dependence, but is often at least partly transient.^88,190,234,377^ Importantly, while there are established protocols regarding other important side-effects, data regarding the management of CIS remain limited, despite the relevance of CIS as a key determinant of treatment adherence.^80,87,88,189,234^ Management of CIS should start with patient education about its potentially transient nature^190^ and about healthy habits regarding sleep hygiene.^190^ It also includes minimizing daytime clozapine administration, avoiding concomitant sedating drugs and clozapine dose reduction whenever possible.^190,234,378^ Importantly, recent findings indicate that clozapine administration partly during daytime may not reduce the burden of sedation.^379^ Emphasizing nocturnal administration whenever possible while taking into account individual preferences appears to be more promising also given that complex dosing instructions might be too demanding for some patients.^379^ Adding aripiprazole constitutes another option.^88,378^ Augmentation with modafinil has also been discussed, but findings from a placebo-controlled pilot trial were not generally supportive.^73,380^ Furthermore, there are reports indicating a pharmacokinetic interaction between clozapine and modafinil, which could lead to a considerable increase of clozapine plasma levels.^381^

## Discontinuation and re-challenge of clozapine

### Managing discontinuation

Approximately 30–40% of patients discontinue clozapine over the course of treatment,^382^ mainly because of side effects, non-compliance to monitoring protocols or patient preference.^234,383^ Both immediate and gradual clozapine termination can result in withdrawal symptoms, especially cholinergic and serotonergic discontinuation syndrome.^276,384–387^

Cholinergic rebound symptoms observed in up to 50% of cases include agitation, delirium, and hallucinations,^386^ vomiting, diarrhea, headache, diaphoresis, dystonia, and dyskinesia.^384,385,388^ Clinical management includes supportive care and treatment with anticholinergic compounds.^386^ Serotonergic rebound symptoms comprise agitation, diaphoresis, clonus, and hyperreflexia. In addition to supportive care, termination of concomitant serotonergic medication might be indicated as well as short-term use of cyproheptadine for moderate and severe cases.^385^ Current evidence supports olanzapine as well as risperidone and long-acting aripiprazole as the best alternative options,^389,390^ but without matching the efficacy of a clozapine re-challenge.^391–393^

Hence, discontinuation of clozapine often leads to a considerable worsening of psychopathology with potentially severe short- and long-term clinical and functional consequences.^217,389,390,394–396^ While withdrawal symptoms also contribute to this deterioration, supersensitivity mechanisms^397–401^ and a lack of sufficiently effective alternative antipsychotics are regarded as the main causes.^384^ Catatonia and persistent psychotic exacerbation are the most common sequelae.^386,397,402^ Here, benzodiazepines and electroconvulsive therapy (ECT) constitute crucial treatment options.^402–404^ There is no evidence for a sufficient medium- and long-term efficacy of ECT without clozapine in most patients with TRS, however.^395,405^

Based on these findings, current guidelines strongly recommend a hyperbolic discontinuation regime, if discontinuation is inevitable.^217,385,406^ Immediate termination of clozapine should be limited to potentially life-threatening side effects including agranulocytosis, myocarditis, ileus or subileus, neuroleptic malignant syndrome (NMS), venous thromboembolism, and diabetic ketoacidosis or hyperosmolar coma.^217^ Most importantly, because of the complications outlined above and its superior efficacy, permanent discontinuation of clozapine should be avoided whenever possible.^217,221^

Rather, the singular status of clozapine underscores the importance to seriously consider a re-challenge even after severe side-effects. This notion is supported by a growing body of evidence for a positive risk–benefit ratio and considerable success rates for clozapine re-challenges after neutropenia,^217,395^ NMS,^407^ and to a lesser extent myocarditis.^224,408,409^ Any re-challenge must only be attempted in an appropriate hospital setting with sufficient support by relevant specialists, that is, cardiologists or hematologists.^224,410^

### Re-challenge after neutropenia

Current evidence clearly argues against a re-challenge after CIA.^269,407^ After CIN, a re-challenge based on a strict risk–benefit assessment is considered to be a reasonable clinical option.^407^ Importantly, time of onset of CIN during a re-challenge is typically shorter.^411^ In general, a slower re-titration rate of clozapine might reduce risk of re-occurrence of CIN.^221^ Based on published cases, the success rate is estimated at about 66%.^407^ Current evidence indicates that concomitant treatment with lithium markedly increases success rates.^269,412,413^ Plasma lithium levels above 0.4 mmol/L have consistently been associated with increased neutrophil counts, most likely due to bone marrow induction.^269,414–416^ Lithium treatment should be initiated at least two weeks prior to a clozapine re-challenge and maintained long-term,^217^ because its discontinuation increases the risk for a re-occurrence of blood dyscrasia.^412^ While it is overall considered to be safe, concomitant treatment with clozapine and lithium can markedly decrease the seizure threshold.^151,417,418^

Treatment with G-CSF is an alternative approach, which increases re-challenge success rates.^287,291,419,420^ To this end, G-CSF can be administered on a regular prophylactic basis during a re-challenge irrespective of current absolute neutrophil count (ANC).^421^ Alternatively, G-CSF can be administered on an as-required basis in the event of predefined neutrophil counts to maintain clozapine treatment. Current evidence indicates that both approaches are safe and effective.^421^ Importantly, currently available evidence indicates that long-term use of G-CSF is not associated with significant morbidity in other patient populations.^422^ Therefore, such a strategy has been proposed for patients in which discontinuation of G-CSF results in recurrent neutropenia.^421^ Long-term safety and efficacy data are currently lacking, however.^419,421,423–426^

To summarize, lithium can be suitable for persistent forms of mild neutropenia, while more severe forms of neutropenia require the use of G-CSF.

### Re-challenge after myocarditis

At present, there are no established consensus guidelines for re-challenges after CIM, but rather protocols based on case series.^243,309,315,407,410,427–431^ Importantly, a re-challenge should only be attempted after patients have made a full recovery from CIM.^427^ It requires intensive clinical, laboratory and electrophysiological (ECG, TTE) monitoring (e.g. every second to third day) and very slow dose titration (e.g. 12.5–25 mg per week),^243,408–410,427,432^ which reduces CIM recidivism risk.^315,427^ Re-challenge success rates after CIM are currently estimated at approximately 60%,^431^ but with a high degree of uncertainty. Notably, compared with re-challenges after CIN, the number of published cases is an order of a magnitude lower.^407^ Therefore, re-challenges after CIM must not be regarded as a routine procedure and warrant exceptional caution.

## Pregnancy and breastfeeding

A switch from a non-clozapine antipsychotic to clozapine might increase fertility as a result of alleviated hyperprolactinemia and amenorrhea.^73^ Currently, there is no evidence for a reduced safety of clozapine for pregnant women.^433^ Several side-effects, however, may be exacerbated including weight gain, constipation, sedation, and orthostatic hypotension.^434^ Close monitoring for emerging gestational diabetes is therefore mandatory.^435,436^ Notably, CYP1A2 enzyme activity decreases by approximately 33% during the first trimester and by approximately 65% during the last trimester.^437^ This necessitates monthly monitoring of plasma clozapine levels throughout pregnancy, repeated a week after delivery.^73^

To date, there is no conclusive evidence for detrimental neurodevelopmental and cognitive long-term effects resulting from fetal exposure to clozapine.^434,438–440^ Clozapine is not associated with greater teratogenic effects than other antipsychotics.^440–442^ Furthermore, there is no evidence for increased rates of prematurity, delivery complications, or changes in birth weight and height compared with other antipsychotics.^434^ Given its unique efficacy for TRS, for the majority of pregnant women receiving clozapine, switching to another antipsychotic is not a feasible option.^434,436^ In most cases, the considerable risk for illness exacerbation associated with such a switch outweighs the risks associated with continued clozapine treatment during pregnancy.^436,439^ Therefore, a thorough risk–benefit assessment is indispensable before any treatment change. Using the lowest effective dose of clozapine is especially crucial during pregnancy, however.^73,433,434,436,438^ Furthermore, frequent gynecological consultations and antenatal screenings should be encouraged and supported.^73,433,434,436,438^

The level of clozapine excretion into breast milk is considerable.^436,438^ Owing to insufficient short- and long-term safety data, breastfeeding during clozapine treatment should therefore be avoided.^434^

## Clozapine treatment in the elderly

There are a number of changes in the pharmacokinetics and pharmacodynamics of clozapine in elderly patients, which warrant particular caution including lower maintenance doses.^73,443,444^ Rates of CIA, CIMs, seizures, and metabolic syndrome increase in older patients.^445–447^ The anticholinergic properties of clozapine may increase the risk for cognitive decline.^91^ Overall, clozapine appears to be both safe and effective in the majority of elderly people when adhering to very low dose titrations and providing increased pharmacovigilance.^443^

## Management of clozapine-resistant schizophrenia

The markedly delayed onset of its full therapeutic effect is a unique characteristic of clozapine, which appears to be only partly attributable to the necessarily slow initial dose titration.^79,448^ Consequently, the current TRRIP consensus guidelines strongly recommend deferring treatment evaluation in accordance with the main target symptoms while also ensuring a plasma clozapine level above 350 µg/L (Table 4).^350^ For predominant positive and mixed (both negative and positive symptoms) symptoms, treatment evaluation after three months is considered to be adequate.^350^ For negative and cognitive symptoms, an evaluation after four months is recommended, while eight weeks are deemed appropriate for aggression and suicidality.^350^

About 40% of patients show an insufficient response to clozapine despite clozapine plasma levels within the recommended range.^25,67,350^ These cases are classified as ultra-treatment-resistant or clozapine-resistant schizophrenia (CRS).^25^ A high genetic load for schizophrenia appears to increase the risk for a poor treatment response.^33,449^ Compared with TRS, CRS is characterized by later clozapine initiation and associated with higher mortality rates.^127,350^ Importantly, clozapine should not be discontinued in patients with CRS, but rather augmented (Table 3).^127,350^ For predominant positive symptoms, recommended augmentation strategies include a combination with amisulpride or aripiprazole as well as ECT.^350,351^ Furthermore, while there is no evidence for clinically meaningful symptom improvements produced by cognitive behavioral therapy (CBT) in CRS, pragmatic individual trials might still be indicated.^450^

## Impact on mortality

Despite its side-effect burden and its detrimental influence on metabolic parameters in particular, clozapine reduces not only suicide mortality but also all-cause mortality to a greater degree than any other oral antipsychotic. Its positive effects on cardiovascular mortality – while less pronounced – are nonetheless comparable with other antipsychotics.^15,46,451^ Clozapine’s mortality reducing effects are likely attributable to its superior efficacy regarding positive symptoms and suicidality, to improved treatment adherence and relapse rates,^46^ as well as to mitigating the increased mortality risk associated with comorbid SUDs.^452^ Consequently, the likelihood of an adequate diagnosis and treatment of somatic comorbidities should also increase.^453^ This might also be facilitated partly by the stricter pharmacovigilance regime required for clozapine. In light of the high rates of somatic comorbidities and the considerable overall increased mortality rates in people with schizophrenia,^454,455^ these findings constitute another crucial argument in favor of a broader use of clozapine while also underscoring its safety.^456^

## Clinical implications

The singular extent of clozapine’s efficacy outlined above has important clinical implications for the treatment of schizophrenia. First and foremost, existing clinical and neurobiological lines of evidence provide little to no reason to be reluctant regarding clozapine use. Consequently, clozapine must never be regarded as an antipsychotic of ‘last resort’ but rather the drug of choice for patients nonresponsive to first-line treatment and more generally for patients with an emerging unfavorable course of illness.^457^ In this regard, stringent use of TRS criteria and close adherence to current recommendations provide excellent guidance for clinical decision-making. The high rates of treatment resistance in first-episode patients underscore the need for early recognition and treatment of TRS.

Slow dose titration is essential to avoid titration-related side effects including sedation, myocarditis, and neutropenia. To this end, dose titration rates of 12.5 mg per day or less during the initial weeks of treatment should be seriously considered. Systematic pharmacovigilance and a timely management of ADRs are very feasible and also among the most important elements of a successful treatment strategy. Furthermore, clozapine might be essential when aiming to address substance abuse and sufficient treatment of somatic comorbidities. Even major pre-existing problems in these areas should not automatically be regarded as obstacles for offering clozapine.^458^ After clozapine initiation, clinicians should exhaust every reasonable option to minimize permanent all-cause discontinuation. This includes carefully re-challenging patients even after serious adverse events like myocarditis and neutropenia. Furthermore, treatment algorithms involving clozapine can and should be very simple. Clozapine use is key for reducing antipsychotic polypharmacy and the associated side-effect burden.^459^ Accordingly, clozapine should always first be used as an antipsychotic monotherapy in both TRS and non-TRS patients. For persistent positive symptoms despite adequate clozapine use, augmentation with an appropriate second antipsychotic should be the first treatment escalation (Table 4), before offering ECT as a second treatment escalation step. Finally, clozapine use requires a long-term approach acknowledging the late onset of clozapine’s full effects. In this context, clinicians also need to be mindful of the fact that some key benefits like reduced suicidality and mortality rates may be next to invisible.

## Conclusion

Underutilization and delayed initiation of clozapine regardless of the continuously mounting evidence and contrary to all recommendations remain a major concern. This underscores the urgent need for intensified research into the real and perceived barriers for clozapine treatment, continued education of psychiatrists, and appropriate structural adjustments of routine clinical care. These efforts are justified not least by the very high patient satisfaction with clozapine despite the considerable complexity of its use and its potential side effects.^10,286,460^ Most importantly however, they are imperative because – going into the sixth decade of its clinical use – compared with all other available pharmacological treatment options, the broad beneficial impact of clozapine on patients’ life remains second to none.

## Declarations

**Ethics approval and consent to participate:** Not applicable.

**Consent for publication:** Not applicable.

**Availability of data and materials:** Not applicable.
